# Observations on the Frequency of Sightings of White Sharks in the Population of Gansbaai, South Africa, in the Presence of Cage Diving

**DOI:** 10.3390/biology14070762

**Published:** 2025-06-25

**Authors:** Primo Micarelli, Francesca Romana Reinero, Antonio Pacifico, Gianni Giglio, Makenna Mahrer, Emilio Sperone

**Affiliations:** 1 Sharks Studies Center—Scientific Institute, 58024 Massa Marittima, Italy; ricerca@centrostudisquali.org (F.R.R.); antonio.pacifico@unimc.it (A.P.); 2 Department of Physical Sciences, Earth and Environment DSFTA, University of Siena, 53100 Siena, Italy; 3 Department of Economics and Law, University of Macerata, 62100 Macerata, Italy; 4 Department of Biology, Ecology and Earth Sciences DIBEST, University of Calabria, 87027 Rende, Italyemilio.sperone@unical.it (E.S.); 5 W. M. Keck Science Department, Claremont McKenna College, Claremont, CA 91711, USA; mmahrer23@students.claremontmckenna.edu

**Keywords:** elasmobranch, South Africa, *Carcharodon carcharias*, ecotourism, cage diving

## Abstract

Shark ecotourism is an ever-expanding reality, with hundreds of thousands of tourists involved annually. Although it represents a fundamental tool for sharing the urgent need to safeguard sharks with the public, the implications for the health and well-being of sharks must be taken into due consideration. In South Africa, cage-diving has been an important form of ecotourism since the 1990s. In Gansbaai, over a 14-year period from 2009 to 2024, excluding the 2020–2021 pandemic years, data was collected from cage diving operators’ boats on the frequency of sightings of white sharks. After approximately 560 h of field observations, averaging about 45 h per year and about 9 h per year between 2023 and 2024, 423 sharks were observed with five re-sightings. We highlighted that the number of sightings did not increase during the presence of cage diving operators and that site fidelity has not been observed by white sharks frequenting this area.

## Simple Summary

In South Africa, white shark tourism has been a popular recreational activity since the 1990s. White shark cage-diving involves a protected and potentially dangerous species, with a general belief by the media that shark behavior and abundance are conditioned by such human activities. However, in South Africa this does not appear to be the case, since no increase in white sharks or fidelity to the site has been observed in over a decade of sightings.

## 1. Introduction

Wildlife tourism is a rapidly expanding industry. In 2015, there were at least 590,000 shark watchers in the shark-related ecotourism sector [1,2,3]. When conducted responsibly, wildlife tourism can provide economic development [2,3] and conservation awareness [4]. Shark-related ecotourism can represent an important support or even alternative to commercial and artisanal fishing, and the conversion of fishermen to this more sustainable activity becomes possible. Evidently this type of ecotourism activity must be carried out with criteria and strategies to mitigate the risk to make the benefits more important than the negative impacts [5,6,7]. Healey et al. [7] identified 151 unique species-activity location conditions for elasmobranchs, categorized by four broad activity types which are diving, snorkeling, provisioning, and cage diving, and they span 42 countries and 49 different species. Each activity type was described as follows:

1. Diving: Tourist activities that utilize SCUBA equipment to view elasmobranchs. Activity does not involve the use of attractants or a shark cage.
2. Snorkeling: Tourist activities involving the use of snorkel equipment to view elasmobranchs. Species interactions will typically occur in close proximity to the surface. Activity does not involve the use of attractants or a shark cage.
3. Provisioning: Tourist activity involving snorkeling, diving, or swimming that uses an attractant, bait, or food rewards for the purpose of aggregating and/or positively reinforcing elasmobranchs to remain in close proximity. Activity occurs outside of or in the absence of a shark cage.
4. Cage Diving: Tourist activities involving the underwater observation of elasmobranchs from a cage. Typically, attractants such as berley and baits are used to lure target species towards the cage. Activity is not restricted to SCUBA diving and may occur through snorkeling or the use of a surface line.

**Figure 1:**
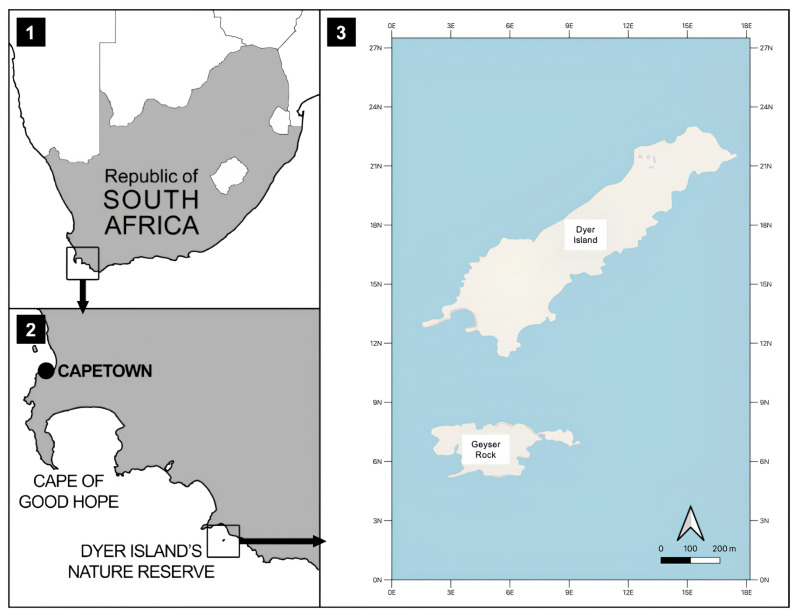
Dyer Island Nature Reserve (Gansbaai, South Africa, 34°41′ S; 19°24′ E).

The most recently available estimates from Cisneros-Montemayor et al. [3] suggest that just in 2011, 590,000 tourists participated in a variety of shark-viewing activities, expending USD 314 million annually across 20 different countries, and it is expected at least to double within the next 20 years. Both governments and the actors directly involved present this type of activity as a useful tool for conservation and for the correct behavior of those who practice it [8]. A useful example is the tiger shark (*Galeocerdo cuvier*, Pèron & Lesueur, 1822) which is a large animal and has a reputation as a dangerous animal at the top of the food chain, making it a popular target for ecotourism in various parts of the world including Fiji, South Africa, and the Bahamas, and in the latter area it has allowed the development of an ecotourism industry that has brought in USD 800 million over twenty years [9,10]. Also, in regard to the white shark (*Carcharodon carcharias*, Linnaeus 1758), thanks to the possibility of observing them live in the field in a safe way, ecotourism activity has been able to develop, although only in some areas of the world [11,12]. These locations include Guadalupe Island in Mexico [13], the Farallon Islands off the coast of California [14], the Western Cape of South Africa [15], the Neptune Islands in Australia [16], and, more recently, the South Island of New Zealand [17]. The white shark is a protected species in many countries such as the United States, South Africa since 1991, New Zealand, Australia, and Mexico [18], and this species has been categorized as Vulnerable since 1996 [18]. This shark is relatively abundant in eight “Great White hot spots” [19,20,21,22,23,24]: central California (in particular the Farallon Islands and Año Nuevo Island), central Chile (from Punta Angamos to Punta Lavapie), New England, the Mediterranean Sea, South Africa (the Indian Ocean side of the Western Cape and the Southern Cape regions), southern Australia, New Zealand, and Japan (from Sendai Bay to Kumano Bay). Among these few areas of the world is also Gansbaai in South Africa, which is an aggregation site for this species and is one of three South African areas, which also includes Mosselbai and False Island, and where it is possible to observe and collect data in the field thanks to the fact that Gansbaai is the only area in the world where it is possible to observe white sharks daily, depending on the weather and sea conditions, thanks to the local ecotourism operators [25]. In Gansbaai, there are eight cage diving operators authorized to carry out activities at sea in delimited areas around Geyser Rock in front of Dyer island (Figure 1) and Joubertsdam beach. Tourist trips last about a couple of hours, while for scientific teams the duration of observations at sea generally varies between 4 and 8 h. Until 2020, before the COVID-19 pandemic, Marine Dynamics annually hosted about 80,000 tourists from all over the world. Since 2022, the presence of tourists has dropped to about 32,000 tourists per year (personal comm. Hennie Otto Marine Dynamics Director). Studies carried out by Towner et al. [26] have indicated that between March and May, in Autumn and from June to August in Summer, it is possible to spot juveniles, sub-adults, and adults, and among these the month of May is the best for direct observations and sightings. Photo-identification is a useful tool during sightings and a valid long-term method for the identification of individual white sharks [27]. One of the benefits of its use is that it is non-invasive although it has the limitation of having to be used by expert personnel. It can also provide information on white shark population size [28,29,30], sexual composition [30,31], longevity [27], site fidelity [27,30,31,32], and migratory behavior [32]. The photo identification must be very detailed, and the database must include data such as sex, length, parasites, and scars on the body to avoid potential mistakes [29,33,34].

Since a potentially dangerous and protected species, such as the white shark, is involved in cage diving activities, this activity has triggered heated journalistic debates [16,35,36]. This ecotourist industry often attracts negative attention following a human/shark incident, with the media presenting the view that sharks are conditioned to humans through cage-diving activities [11,37]. Among the topics of these heated debates are concerns about the welfare of this species and also the possible negative impacts it could have on their behavior, such as an increase in their sightings in areas subject to ecotourism with cage diving. The study carried out by Bruce et al. [16], in the Neptune Islands in Australia, where cage diving operators are active, highlighted an increase in the frequency of sightings, indicating a change in the behavior of white sharks in the long term of a highly vagile animal. On the contrary, studies conducted in French Polynesia for a period of six years have not shown any variations in the frequency of sightings and, in particular, there has not been an increase [38]. The aim of this paper was to observe and record sightings for a duration of 14 years and verify if during the cage-diving activity in Gansbaai (South Africa), where eight cage diving operators work on a daily basis throughout the year, an increased frequency of sightings of the transient white shark population [34] occurred. This work represents the first contribution aimed at evaluating any variations in the sighting frequencies of white sharks in the presence of cage diving operators in Gansbaai and aims to contribute to informing future conservation efforts in the area.

## 2. Materials and Methods

The data collection was performed in South African waters around the Nature Reserve of Dyer Island, which is located 7.5 km off the coast of Gansbaai (34°41′ S; 19°24′ E). This Reserve includes Dyer Island and Geyser Rock (Figure 1): the first is a low-profile island ca. 1.5 km long and 0.5 km wide and is characterized by the presence of different seabird colonies; the second island is ca. 0.5 km long and 180 m wide and it hosts a colony of Cape fur seals *Arctocephalus pusillus* (Schreber, 1775). Dyer Island Nature Reserve is located in a special area known as the Agulhas Bioregion, which has the distinction of being the meeting point of the Benguela Current, known as the eastern boundary current of the subtropical vortex located in the South Atlantic Ocean and where it meets the Agulhas Current, which in turn represents the western limit of the Indian Ocean. In the summer, the intensified southeast trade winds cause the cold waters of the Benguela Current to rise and enter the bay [39]. In this way, upwelling induces significant biological productivity, which allows the maintenance of significant fish stocks of sardines, anchovies and hake [40], which together form the basis of the white shark’s food chain.

Fourteen expeditions were conducted in the study area each year between 2009 and 2024, with the exception of the COVID-19 pandemic years (2020–2021), to study social and predatory behavior in presence of passive preys [33,41,42] and sighting frequency across the years. The observations were carried out between 2009 and 2013 using a 12-m vessel, the Barracuda, owned by Shark Diving Unlimited, and between 2014 and 2024 using the 14-m long Slashfin, owned by Marine Dynamics, both ecotourism companies active in Gansbaai, and the activities took place during the South African autumn between March and May. The water temperatures recorded in these months ranged from 13.5 °C to 18 °C, and underwater visibility varied from 2 to 5 m (measured to the nearest 0.5 m using a Secchi disc). The rectangular floating cage, used to observe the sharks in the water, was made of galvanized steel. The cages could accommodate up to three researchers at a time and were attached to the side of the vessel with strong lines. Sightings of white sharks occurred in two distinct aggregation areas in Gansbaai: Geyser Rock Island and in front of Jouberstdam Beach, as also described by Towner et al. [29]. The techniques used to attract sharks were the same as those previously employed by Laroche et al. [41] using olfactory stimulants (Chum). The olfactory stimulants or chum were characterized by a mixture containing sea water, fish blood and sardines, and cod liver oil and was mainly made up of liquids. Tuna heads of about 2–3 kg each were also used as bait and kept afloat with the help of special floats [42]. Despite some incidental feeding events by the sharks on the bait, the research did not fall under the definition of provisioning. Sharks were not directly fed but rather attracted by chumming and tuna heads attached to ropes. When the sharks got adequately close to the cages, the tuna heads were pulled back on board by operators.

The observations made from the boats lasted from 6 to 8 h/day, whereas underwater observations lasted between 2 and 4 h/day, for a total of ca. 45 h per year [34]. Expeditions went from 08:00/09:00 in the morning until 14:00 or 16:00, depending on the years and environmental conditions. The identification of individual specimens was obtained through photo-ID [34,43]. The sharks’ data were always collected by the same operators and dorsal fin photos were collected using three different digital cameras across all expeditions: one CANON model EOS 550 with a SIGMA 70 lens (Canon INC, Ota City, Japan), one CANON model 70D with an 18–125 mm lens, and one CANON model 700D with a 55–250 mm lens. All the images were then analyzed with Photoshop (Version 22.5.2) and Excel (Microsoft Excel 97-2003) which was used to archive photographs [34]. The identification of individuals was based on pattern recognition, which included not only the different notches of the dorsal fin [27], but also the following characteristics: caudal and pelvic fin patterns, presence or absence of claspers, gill slashes and body patterns, and presence of scars and ectoparasites. Photo-ID provided the benefits of tracking total shark numbers in the area over time, determining individual long-term site use, and might also facilitate further analysis such as network analysis or mark-recapture studies [43,44], which were not undertaken in the current study. Each collected photo was then numbered and tagged with the following information: date, sex, size of the shark, and weather cnditions upon observation [43]. Not all white sharks sighted were recorded as individual specimens; some sightings were identified as sharks that had been observed in a previous year, denoted as a ‘resighting.’ The notches on the back line of the dorsal fin represent a sort of fingerprint that does not change over time and were therefore the only characteristic used to evaluate re-sightings of the same individuals in different years. 

Chi-square test of independence was used and applied in 2024, to verify that the frequency of sightings was not linked to the presence of cage-diving operators and that sharks did not exhibit site fidelity behavior. The Chi-square test of independence is a statistical hypothesis test used to determine whether two variables are likely to be related (dependence) or not (independence). This test is very useful when studying variables characterized by frequency counts. More precisely, the statistic test is obtained solving the ration between the mean squared error from the theoretical frequencies and their weights.

## 3. Results

**Figure 2:**
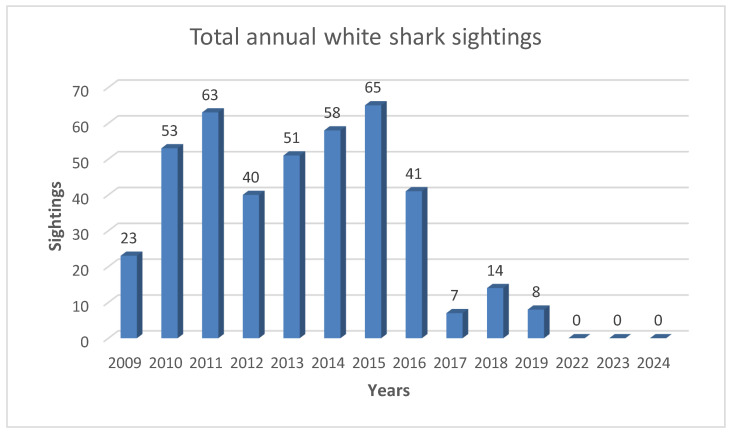
White shark (*C. carcharias*) sightings annually during data collection activities in Gansbaai (2009–2024).

**Figure 3:**
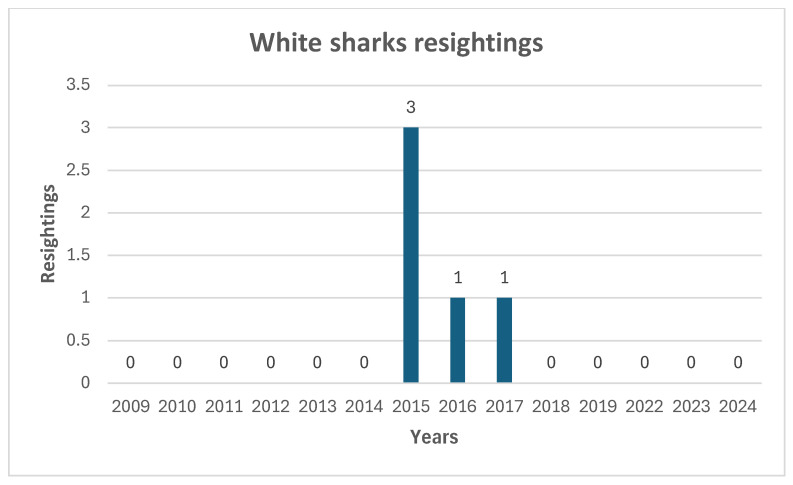
Annual great white shark resightings (*C. carcharias*) in Gansbaai (2009–2024).

During the fourteen years of data collection, 423 white sharks were sighted (Figure 2), for a total of 560 h of field observations. These observations averaged out to about 45 h per year, with the exception of 2023–2024, which both had an average of 9 observation hours per year. Just five sharks were resighted in at least one other year (Figure 3).

The frequency of sightings was not linked to the presence of cage-diving operators. In this study, the independence between sightings ($\chi_{S}^{2}$) and re-sightings ($\chi_{RS}^{2}$) with cage-diving operators were tested. According to the $\chi^{2}$’s test statistics, the null of independence cannot be rejected (*p*-value bigger than the significance level set to 5%).

(1) $$\chi_{S}^{2}=104\;and\;p\text{-}value=0.15\;\left(H0\right)$$

(2) $$\chi_{RS}^{2}=43.556\;and\;p\text{-}value=0.18\;\left(H0\right)$$

Sharks do not exhibit site fidelity behavior. Running the chi-square test statistics to verify that the sharks do not show site fidelity behaviors, the null of independence is not rejected at 5%. More precisely, the test statistic is conducted between the number of sightings and re-sightings with the sighting frequency.

(3) $$\chi_{S}^{2}=91\;and\;p\text{-}value=0.28\;\left(H0\right)$$

(4) $$\chi_{RS}^{2}=23.833\;and\;p\text{-}value=0.30\;\left(H0\right)$$

## 4. Discussion

During the fourteen years of observations carried out between 2009 and 2024 in Gansbaai, an increase in the frequency of white shark sightings was not observed. Indeed, as already indicated by Ferreira and Ferreira [20], increasing peaks over 4 years with subsequent drops in sightings were observed. Additionally, in terms of site fidelity, only five specimens were observed to be present in more than one year over the course of these expeditions. During this observation period, but starting from 2017, we observed a decrease in sightings that could be linked both to the presence of a pair of transient killer whales in the region (Orcinus orca, Linnaeus 1758) [34,45], and in conjunction with the effects of climate change [26,46,47]. Laroche et al. [41] conducted a similar study from June to October 2004 which examined the effects of provisioning ecotourism on the behavior of white sharks around a seal colony in False Island, South Africa. Although ecotourism activity had an effect on the behavior of some sharks in the Laroche study, this effect was relatively minor, and the majority of sharks showed little interest in the food rewards on offer. He considered it unlikely that conditioning would occur from the amount of ecotourism activity tested because the sharks that accounted for most of the data presented in that study showed a nearly ubiquitous trend of decreasing response with time. Laroche et al. [41] also stated that only three operators were granted permits to attract sharks at Seal Island while another location on the South African coast (Dyer Island, Gansbaai) supports eight operators, raising the possibility that the amount of chum used in their study simply was not strong enough to elicit the changes in shark behavior that might occur due to current ecotourism activities. Towner et al. [29] also indicated that, while it may be possible that chumming and diving activities have changed white sharks’ behavior and residence times [16], the opposite may also be true [41]. In the above-mentioned case of French Polynesia, despite the feeding activity, during the six years of observation no evidence was recorded that this type of activity leads to an increase in site fidelity or resident behavior of tiger sharks present in this ecotourism site [38]. This conclusion was consistent with the present study in Gansbaai, where the cage-diving activities were comparatively less disruptive due to the lack of direct provisioning. It is also worth noting the point made by Seguigne et al., in particular that the observed behavioral patterns of tiger sharks were best explained by natural movements, including general wandering within home ranges along the coast and seasonal migrations [38]. It is important to note instead that Bruce and Bradford [48] stated that the results of a study conducted around the Neptune Islands (Australia) demonstrated that cage-diving may lead to long-term, albeit localized, changes in the behavior of a highly vagile shark species. Even if the white shark visitation to the Neptune Islands was temporary and individual sharks varied in their time spent at the site, the level of interaction with the shark cage-dive operators (SCDOs) indicates the effects of the activity were unlikely to be uniform [49]. The researchers suggest that further research is required to understand and minimize any impacts the SCDOs may have on the behavior of white sharks [16,49]. Rogers [50] asserted that white shark residency increased marginally at the North Neptune Islands and declined at the South Neptune Islands in 2015–2016. Across those same islands, Huveneers et al. [51] provides evidence of the effect of wildlife tourism on the activity of a marine apex predator and potential implications for its daily energy budget. That study also affirms that our understanding of behavioral responses to anthropogenic influences must be improved and that future research should both quantify the amount of time white sharks interact with cage-diving operators and estimate its effect on white sharks in relation to their daily energy budget. We agree with this proposal to be a future line of research in Gansbaai. Exposing humans to sharks via shark-based tourism has the potential to help provide an economic alternative for fishermen and reverse the negative public image of sharks by presenting a more realistic image. This narrative reframing is probably the most significant contribution that tourism can offer for shark conservation [52], if made according to precise standards.

## 5. Conclusions

In Gansbaai, over a 14-year period from 2009 to 2024, minus the COVID-19 pandemic years, 423 sharks were sighted, with five re-sightings over 560 h of field observations. The frequency of sightings does not seem to be linked to the presence of cage-diving operators as it has not increased from 2009 to 2024. Sharks frequenting this area do not exhibit site fidelity behavior and the presence of cage-diving operators does not seem to contribute to alter the behavior of white sharks as observed on Neptune Island in Australia. Additionally, in the South African area, further observations are desirable to evaluate any effects on the energy balance of white sharks while in the presence of cage-diving boats, particularly because the white shark population in this area appears to have been in serious ecological disarray since 2017, an observation which warrants further investigation.

## Data Availability

The raw data supporting the conclusions of this article will be made available by the authors on request.
